# Review of the Amphibian Immune Response to Chytridiomycosis, and Future Directions

**DOI:** 10.3389/fimmu.2018.02536

**Published:** 2018-11-09

**Authors:** Laura F. Grogan, Jacques Robert, Lee Berger, Lee F. Skerratt, Benjamin C. Scheele, J. Guy Castley, David A. Newell, Hamish I. McCallum

**Affiliations:** ^1^Environmental Futures Research Institute and School of Environment and Science, Griffith University, Nathan, QLD, Australia; ^2^University of Rochester Medical Center, Rochester, NY, United States; ^3^One Health Research Group, College of Public Health, Medical and Veterinary Sciences, James Cook University, Townsville, QLD, Australia; ^4^Faculty of Veterinary and Agricultural Sciences, Melbourne Veterinary School, University of Melbourne, Werribee, VIC, Australia; ^5^Fenner School of Environment and Society, The Australian National University, Canberra, ACT, Australia; ^6^Threatened Species Recovery Hub, National Environmental Science Program, Fenner School of Environment and Society, The Australian National University, Canberra, ACT, Australia; ^7^Forest Research Centre, School of Environment, Science and Engineering, Southern Cross University, Lismore, NSW, Australia

**Keywords:** chytridiomycosis, immune, innate, adaptive, frogs, declines, amphibian, *Batrachochytrium dendrobatidis*

## Abstract

The fungal skin disease, chytridiomycosis (caused by *Batrachochytrium dendrobatidis* and *B. salamandrivorans*), has caused amphibian declines and extinctions globally since its emergence. Characterizing the host immune response to chytridiomycosis has been a focus of study with the aim of disease mitigation. However, many aspects of the innate and adaptive arms of this response are still poorly understood, likely due to the wide range of species' responses to infection. In this paper we provide an overview of expected immunological responses (with inference based on amphibian and mammalian immunology), together with a synthesis of current knowledge about these responses for the amphibian-chytridiomycosis system. We structure our review around four key immune stages: (1) the naïve immunocompetent state, (2) immune defenses that are always present (constitutive defenses), (3) mechanisms for recognition of a pathogen threat and innate immune defenses, and (4) adaptive immune responses. We also evaluate the current hot topics of immunosuppression and immunopathology in chytridiomycosis, and discuss their respective roles in pathogenesis. Our synthesis reveals that susceptibility to chytridiomycosis is likely to be multifactorial. Susceptible amphibians appear to have ineffective constitutive and innate defenses, and a late-stage response characterized by immunopathology and Bd-induced suppression of lymphocyte responses. Overall, we identify substantial gaps in current knowledge, particularly concerning the entire innate immune response (mechanisms of initial pathogen detection and possible immunoevasion by Bd, degree of activation and efficacy of the innate immune response, the unexpected absence of innate leukocyte infiltration, and the cause and role of late-stage immunopathology in pathogenesis). There are also gaps concerning most of the adaptive immune system (the relative importance of B and T cell responses for pathogen clearance, the capacity and extent of immunological memory, and specific mechanisms of pathogen-induced immunosuppression). Improving our capacity for amphibian immunological research will require selection of an appropriate Bd-susceptible model species, the development of taxon-specific affinity reagents and cell lines for functional assays, and the application of a suite of conventional and emerging immunological methods. Despite current knowledge gaps, immunological research remains a promising avenue for amphibian conservation management.

## Introduction

Two decades have passed since the discovery and characterization of the devastating amphibian skin disease, chytridiomycosis, caused by two unusual fungal species that likely originated in Asia (1, 2). *Batrachochytrium dendrobatidis* (hereafter Bd) was first detected in the 1990s and is now widespread globally (3, 4), whereas *B. salamandrivorans* (Bsal) primarily affects salamanders (5) and was recently described after arriving in Europe in 2010. Chytridiomycosis is reported to affect >350 amphibian species and has had a dramatic worldwide impact on amphibian biodiversity, having caused the decline and possible extinction of greater than 200 species (6, 7). Chytridiomycosis has thus been the subject of intense study, with research focused on understanding fungal virulence, pathogenesis, immunology, treatment, and epidemiology [reviewed in (8–11)]. A central aim of this research has been finding ways to mitigate disease in the field to reduce or prevent further species declines and extinctions (12, 13).

The evolution of resistance and/or tolerance to infection is a key long-term goal for managing *in situ* amphibian populations in regions where Bd is now enzootic (14), and immunological research is central to this goal. A recent study demonstrated that the fungus can maintain high virulence post-emergence (15), which may be a result of its broad host range (where fungal persistence may not be affected by the loss of highly susceptible host species). However, many amphibian species are recovering in the wild (10), and some have increased survival rates consistent with improved immunity (16). A study comparing skin secretion inhibitory activity against Bd pre- and post- emergence suggests that the evolution of natural immunity may be occurring in some species *in situ* (15). Several studies have made progress uncovering other putative mechanisms for improved immunity, including directional selection of major histocompatibility complex (MHC) alleles (17–21). Unfortunately, many endangered frog species appear to be running out of time. Without sufficient genetic, phenotypic, or behavioral evolution of the host, many susceptible populations remain threatened by chytridiomycosis and are experiencing ongoing declines, sometimes decades post-initial chytridiomycosis outbreaks (10, 22–24). Other susceptible species may persist despite chytridiomycosis-associated mortality due to high reproductive capacity. However, compensatory recruitment may be reducing selection pressure for the evolution of immunity (25), and these populations remain highly vulnerable to other threats (26). Furthermore, animals repatriated from captivity continue to succumb to disease in the field (27, 28).

While the amphibian immune response to chytridiomycosis has been the subject of some research to date, many aspects remain poorly understood, likely owing to the complexity of the system and the vast range in species' responses to infection. Indeed, Bd and Bsal are the main fungi from their phylum found to cause disease in vertebrates, and the observed host immune response to these pathogens appears to depart from an expected “normal” immune response to an intracellular or fungal pathogen. Previous reviews [e.g., (11, 29–31) have covered (1) components of innate immune defenses such as secretion of skin antimicrobial peptides, and maintenance of symbiotic skin bacteria and their antifungal metabolites (29, 32), and (2) adaptive immune components such as MHC allele selection, antibody production, and lymphocyte responses (33, 34). However, the field is overdue for an update that incorporates the results of recent transcriptomic and immunogenetic studies, as well as to provide a more thorough overview of the role of key immune components. Concerning the innate arm of the immune system, virtually nothing is known about the role of pattern recognition receptors (PRRs), complement, cytokines and chemokines, macrophages and dendritic cells, other phagocytes, and natural killer cells. For the adaptive arm of the immune system, besides the possible inhibition of lymphocyte proliferation response by Bd and importance of antibodies in the skin of infected frogs, very little is known about B and T cell responses, immunological memory and antigen detection. Improving our capacity for amphibian immunological research and our understanding of the host immune response to chytridiomycosis may result in numerous applied benefits. These may include: (1) identifying targets for further research, treatment, and marker-assisted evolution, (2) identifying immunologic management strategies including environmental manipulation, vaccine design, selective breeding, genetic engineering and pathogen virulence attenuation, and (3) predicting species at continued risk of decline and implementing timely mitigation measures.

**Figure 1:**
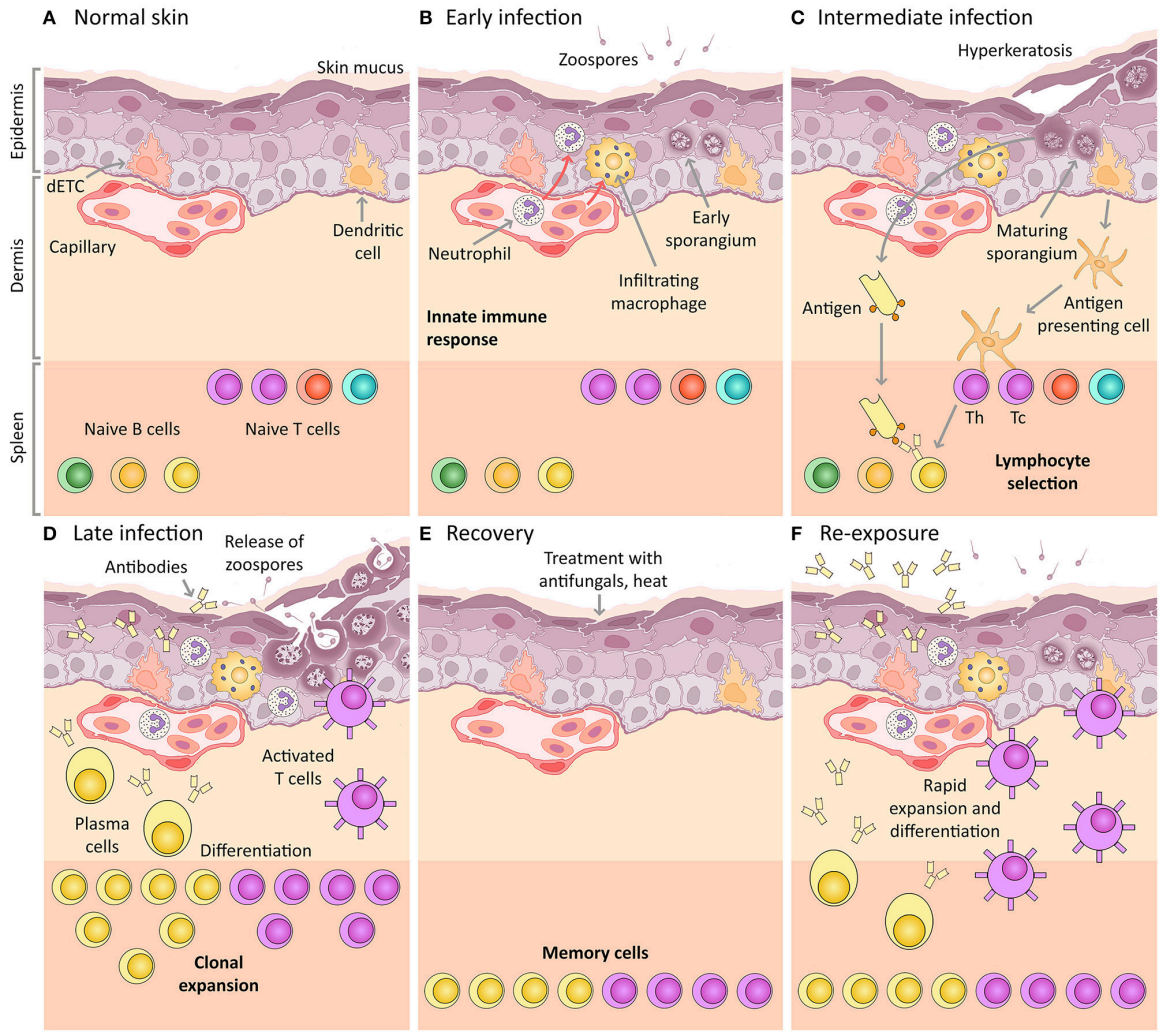
Amphibian host immunity schematic, depicting a histological section through the skin and progressive infection stages with Bd. The inference of the main cellular components is based on mammalian immunology and an expected “normal” immune response (including the expected response to vaccination). **(A)** Normal skin: Layers of uninfected frog skin epidermis including, from deepest to most superficial, the *basal lamina, stratum germinativum, stratum spinosum, stratum granulosum, stratum corneum* and the superficial mucus layer. Two immune surveillance cells are illustrated within the epidermis, an immune dendritic cell (homologous to Langerhans cell), and a dendritic epidermal T cell (dETC). Within the dermis is a capillary with the nucleated red blood cells of amphibians. An example complement of naïve B and T lymphocytes are depicted waiting quiescent in the spleen, illustrated schematically as the lower band on the figure (please note that the spleen is a separate organ and does not lie adjacent to the dermis in living amphibians). **(B)** Early infection: Expected immune mechanisms upon initial exposure to Bd, assuming constitutive defenses (such as AMPs and bacteria) are insufficient. Zoospores are illustrated penetrating the mucus layer, and early thalli with zoosporangia developing are illustrated inside deeper host cells. In a normal immune response, pathogen recognition should lead to the infiltration of innate immune cells, illustrated here to include macrophages and granulocytes (such as neutrophils). **(C)** Intermediate infection: Expected response at an intermediate stage of infection includes the recognition of antigens by dendritic cells that then differentiate into antigen presenting cells and migrate to the spleen enabling antigen-specific selection of lymphocytes. Simultaneously, membrane-bound immunoglobulin on naïve B lymphocytes is exposed to extracellular Bd antigens (transported via the blood circulatory and lymphatic systems). With the assistance of T helper cells, these B cells are activated to respond to infection. **(D)** Late infection: The late adaptive response involves lymphocyte clonal expansion, differentiation into plasma cells and activated T cells (including cytotoxic and helper T cells), as well as the production of antibodies by plasma cells. **(E)** Recovery: If the frog is cleared of infection (perhaps by topical antifungals or heat), the skin might be expected to return to normal, however, a cohort of selected memory lymphocytes should remain. **(F)** Re-exposure: If the frog is then later re-exposed to Bd, the memory lymphocytes (produced during the previous clonal expansion) are then activated and induced to replicate and differentiate, leading to a much more rapid and effective adaptive immune response on re-exposure. This is the concept of immunization (vaccination).

In this review, we present an integrated synthesis of current understanding of the amphibian host immune response to chytridiomycosis within the classical scaffold of innate and adaptive immunological mechanisms [reviewed in (35)]. We have targeted this review for amphibian chytridiomycosis researchers, but we expect it will also be of interest for researchers in the broader fields of fungal immunology and amphibian conservation. We focus specifically on host mechanisms; predominantly in response to Bd [host responses to Bsal are likely similar but are currently poorly understood; reviewed in (11)]. We do not attempt to review the vast range of factors contributing to variations in susceptibility to infection between individuals and species. For a broad introductory overview of chytridiomycosis, see Box 1. For convenience, we provide a glossary of terms and abbreviations in Box 2. Throughout this review, where amphibian-specific immune knowledge is lacking, we instead refer to the better characterized immune system of mammals. Please see Box 3 for a brief comparison between amphibian and mammalian immune systems. We also focus primarily on post-metamorphic and adult amphibians (especially anurans) because larval amphibians (tadpoles) usually survive Bd infections that localize to their keratinized mouthparts (see Box 4 for a brief overview of tadpole vs. post-metamorphic immune systems). We start by outlining several key (non-mutually exclusive) immune stages: (1) the naïve immunocompetent state, (2) immune defenses that are always present (constitutive defenses), (3) mechanisms for pathogen recognition and induction of innate immune defenses, and (4) adaptive immune defenses. For each stage, we briefly describe the expected immune response to an invading infectious organism such as Bd (see Figure 1), then compare it with current knowledge of chytridiomycosis, highlighting research gaps. We then examine and discuss evidence for the role of immunosuppression and immunopathology in chytridiomycosis. We conclude by suggesting future research directions that will contribute to improving mitigation strategies for chytridiomycosis.

Box 1 **Amphibian chytridiomycosis–pathogens, infection and disease basics.**

- The *Chytridiomycota* are a phylum of microscopic predominantly saprobic fungi with a biphasic life-cycle consisting of motile flagellated zoospores and reproductive zoosporangium (36). Two species within the *Chytridiomycota* have been well characterized and shown to parasitise vertebrate hosts (*Batrachochytrium dendrobatidis* (Bd) and *B. salamandrivorans* (Bsal)). These species both infect amphibians causing the disease chytridiomycosis (3–5). The pathogens replicate intracellularly within the deeper cell layers of the host epidermis. In tryptone-gelatin hydrolysate-lactose (TGhL) broth medium at 22°C, Bd takes 4–5 days to mature from zoospore to mature zoosporangium (37–39).
- Infections with Bd and Bsal are restricted to keratinized epidermis of amphibians (mouthparts of anuran tadpoles, skin of adult amphibians). Chytridiomycosis causes a range of changes in the host epidermis including hyperkeratosis, hyperplasia, ulceration, erosions, and necrosis (5, 37). In severe infections, clinical signs may include lethargy, abnormal posture, anorexia, peripheral erythema, increased skin shedding (40, 41) and mortality usually over a period of 2–6 weeks post-exposure. *Batrachochytrium salamandrivorans* infects deep epidermal layers and is more erosive and ulcerative without demonstrating a hyperplastic response. Tadpoles infected with Bd may exhibit blunting of mouthparts and sublethal effects on growth and development (42), but the infection is not usually fatal until metamorphosis and widespread keratinization of the skin (43–45).

Box 2 **Abbreviations and glossary of terms.**

- Adaptive immune system: the arm of the immune system mediated by B and T cells, characterized by specificity, immunological memory and recognition of non-self antigens.
- Agglutination: the combination of antibody and antigens forming an aggregate
- Allele: one form of a gene at a single locus
- Antibody (immunoglobulin): glycoprotein molecules capable of reacting specifically and selectively with antigens
- Antigen: a substance that reacts with the products of an immune response (antibodies)
- Antimicrobial peptides (AMPs): small, generally cationic and relatively hydrophobic peptides that have the capacity to damage bacterial and fungal cells
- Anorexia: lack or loss of appetite for food
- Anura: the Order of tailless amphibians that includes frogs and toads
- Antigen presenting cell (APC): a cell that processes and presents antigen in conjunction with major histocompatibility complex molecules
- Apoptosis: programmed cell death
- B cell: a type of lymphocyte capable of synthesizing antibody (immunoglobulin) in response to an antigen, and possessing B cell receptors (BCR)
- Cationic: positively charged ion
- Caudata (or Urodela): the Order of tailed amphibians that includes salamanders and newts
- Chemotaxis: directional movement of a cell in response to a substance gradient
- Codominance: the full expression of both alleles in a gene pair of a heterozygote
- Complement: a set of blood proteins that enhance the ability of antibodies and phagocytic cells to clear pathogens
- Constitutive defenses: forms of defense that are always present, rather than induced by the presence of a stimulus
- Co-stimulation: cell activation requires stimulation by both an antigen and additional molecules
- Damage associated molecular patterns (DAMPs): host molecules that stimulate a non-infectious inflammatory response
- Dendritic cells: cells with a branched structure that act as professional antigen-presenting cells
- Dermis: the skin layer beneath the epidermis that contains blood capillaries, glands and nerve endings
- Ecdysis: the process of shedding/sloughing old skin
- Endocytosis: mechanism whereby substances are taken into a cells via membrane vesicles
- Epibiotic: living on the surface of another organism
- Epidermis: upper layers of skin containing keratinocytes
- Epitope: a small area on an antigen that can combine with an antibody
- Erythema: redness of the skin
- Humoral: immunity based on antibodies
- Hydrophobic: insoluble in water
- Immunocompetent: ability to respond immunologically to a stimulus
- Immunosuppressed: decreased ability to respond immunologically to a stimulus
- Innate immune system: the arm of the immune system characterized by nonspecific responses not requiring previous exposure to similar antigen
- Langerhans cells: phagocytic cells within the epidermis that function as antigen presenting cells
- Ligand: a molecule that forms a complex with another molecule
- Lymphocytes: cells principally of the adaptive immune system consisting of B and T cells
- Lyse: rupture of cell membrane
- Membrane attack complex (MAC): the terminal part of the complement system that comprises 5 proteins that associate together and cause damage to membranes
- Macrophage: a large phagocytic cell found in tissues, derived from monocytes in blood
- Mitogen-activated protein kinase (MAPK): a protein kinase specific to serine/threonine
- MBL-associated serine protease (MASP)
- Mannose-binding lectin (MBL): a C-type lectin that serves as a pattern recognition receptor and when engaged by pathogen molecules, activates the lectin pathway of complement activation
- Major histocompatibility complex (MHC): a chromosomal locus composing multiple genes encoding histocompatibility antigens (cell surface glycoproteins), includes genes encoding both class I and II molecules
- Neutrophil: a type of white blood cell with phagocytic properties and a segmented nucleus
- Nuclear factor kappa-light-chain-enhancer of activated B cells (NFκB)
- Opsonization: coating the surface of antigens or microorganisms with opsonins to facilitate phagocytosis
- Pathogen-associated molecular patterns (PAMPs): molecular motifs broadly expressed by pathogens and not found on host tissues
- Peptidoglycan recognition proteins (PGRPs): a group of pattern recognition receptors capable of recognizing peptidoglycan wall of bacteria
- Phagocytosis: the uptake of particulate materials by a cell for destruction
- Polymorphism: more than one allele occupies a gene's locus within a population
- Pattern recognition receptors (PRR): receptors that recognize molecular patterns of microorganisms
- Rhizoids: filamentous outgrowth
- Saprobic: lives on decaying organisms
- Somatic hypermutation: a programmed process of mutation within the variable regions of immunoglobulin genes
- Somatic recombination: alteration of DNA of a somatic cell
- T cell: a type of lymphocyte that plays a central role in cell-mediated immunity, and possesses T cell receptors (TCR)
- Tumor necrosis factor alpha (TNF-α)

Box 3 **The immune system of amphibians is similar to other vertebrates.**

The larval and adult immune system of amphibians, in particular *Xenopus* spp. (South African clawed frog), has been subject to extensive investigation as a transitional non-mammalian model organism for comparative and evolutionary immunology and studies of immune ontogeny (46). The adult anuran immune system is fundamentally similar to other jawed vertebrates (47), and responds similarly to antigenic stimulation (48). However, there are some differences.

- Most anurans lack the lymph nodes of mammals (49), and instead rely on the other major lymphoid organs. The spleen represents a primary lymphoid organ (site of lymphopoiesis) and secondary lymphoid organ (site of antigen presentation, T and B cell antigen-dependent activation and expansion) in amphibians. The thymus and liver are also sites of lymphopoiesis, with lymphocyte aggregations additionally occurring in the liver, kidneys, and intestine (50).
- Many amphibians produce potent antimicrobial peptides in granular (serous) glands of the skin [reviewed in (29)].
- Amphibian innate immune cell types are morphologically similar to those of mammals and include polymorphonuclear cells (neutrophils, eosinophils, and basophils), as well as monocytes, macrophages and natural killer cells (46).
- Many innate immune genes and gene pathway homologs to other vertebrates have been identified in *Xenopus* spp. (46). These include receptors [polymorphic major histocompatibility class I and II genes, and toll-like receptors; (51)], cytokines (interferon-γ, interleukins, tumor-necrosis factor α), and complement [classical, alternative, and lectin pathways; (52)].

Although many aspects are still poorly characterized in amphibians (particularly CD4 T helper cell function), adaptive B and T cell biology is fundamentally conserved between mammals and amphibians. However, there are several notable differences.

- Concerning B cell response, the affinity maturation of antibody is relatively poor (10x) in comparison with that in mammals (10,000x), which may be related to the lack of germinal centers important for the selection of B cells expressing antigen receptors with higher affinity (50, 53–55).
- The recent characterization of dendritic cells in the spleen that perform the additional duty of follicular dendritic cells (specialized cells critical for antigen-specific B cell activation), further suggests a less powerful B cell response capacity in amphibians (56, 57).
- Furthermore, unlike mammalian lymphocytes, many differentiated B cells have phagocytic capabilities (58).
- The antibody responses of adult amphibians also differ slightly from mammals and consist of IgM, IgX (mainly mucosal expression) and IgY (induced via T-cell dependent responses); the latter two are functionally analogous to IgA and IgG of mammals (49). Two further isotypes have also been discovered mainly in the spleen, IgD, and IgF (59, 60). IgD is homologous to mammal and fish IgD, although IgF does not have a known mammalian homolog (61).
- Similarly to the situation with B cells, while the CD8 T cell response is MHC class I-restricted and critical for host resistance to viral infection (62), CD8 T cell expansion appears to be not as extensive as in mammals (63).
- Finally, a prominent immune surveillance system based on a large family of MHC class I-like genes regulating the development and function of innate-like T cells critical for host antimicrobial defenses has recently been unveiled in *Xenopus* spp. (64–66). Similar systems are likely to exist across all aquatic vertebrates (67), which will require full consideration in the context of Bd host responses as well as when analyzing transcriptomics (e.g., issues in distinguishing classical MHC and MHC-like transcripts).

Box 4 **Comparison between tadpole and post-metamorphic amphibian immune system.**

- The immune system of tadpoles, while competent, is functionally less well developed than the immune system of post-metamorphic and adult amphibians.
- The amphibian immune system undergoes substantial remodeling accompanied by immunosuppression during metamorphosis, through until about 6 months post-metamorphosis (46, 68, 69).
- The immunoglobulin repertoire is typically smaller and less specific in tadpoles, the thymus involutes and is re-formed during metamorphosis, and the expression of MHC class I and II molecules greatly expands (46, 47, 70).
- The combined effects of (1) immune system remodeling, and (2) the development of keratinized epidermis across the body during metamorphosis, may help to explain why newly metamorphosed anurans are particularly vulnerable to chytridiomycosis (30, 43, 71).

## The immunocompetent uninfected state

Normal uninfected integument of an immunocompetent amphibian host consists of epidermal and deeper dermal layers (Figure 1A). The epidermis constitutes an immediate innate physical defense barrier against pathogen invasion and consists of cell layers that mature as they migrate toward the skin surface. Epidermal layers include the *basal lamina* (basement membrane), then the roughly cuboidal or columnar-shaped proliferative cells of the *stratum germinativum* followed by the *stratum spinosum* and the *stratum granulosum*, through to the highly differentiated keratinized squamous epithelial cells found at the surface of the skin, the *stratum corneum*. These superficial cells are joined by tight junctions, which help maintain the skin barrier (35, 72). Intermittent sloughing of the outer layer of the epidermis, the *stratum corneum*, may assist in the physical removal of skin microorganisms (73). On the surface of this uppermost stratum sits a layer of mucus produced by mucous glands. This mucus contains a number of defensive molecules, including (1) lysozyme and other enzymes produced by phagocytes and keratinocytes, (2) antimicrobial peptides secreted via serous glands, (3) mucosal antibodies, as well as (4) commensal symbiotic bacterial communities together with their secreted antimicrobial compounds [reviewed in (11, 35)]. A number of peripheral immune surveillance cells are typically present in the epidermis, particularly epidermal dendritic cells including putative Langerhans cells (74), which in *Xenopus* spp. express high levels of MHC class II molecules (75, 76). These various dendritic cells are likely to serve as efficient antigen presenting cells, although this remains to be demonstrated. Dendritic epidermal T cells (DETCs), and gamma/delta T cells have also been described in *Xenopus* spp. (76).

The highly collagenous dermis underlies the epidermis. It consists of deeper *stratum compactum* and thicker and more superficial *stratum spongiosum*, separated in some species by the *substantia amorpha* granular calcified layer (77–79). The dermis provides nutrition and sensory integration to the epidermis via a network of capillaries and nerves that course through the dermis. Serous (granular or poison) glands and mucous glands are also present within the dermis, along with pigment-bearing chromatophores and smooth muscle fibers. Serous glands are often widely distributed throughout the skin and are able to discharge their contents in response to noxious stimuli. In the uninfected state, the repertoire of naïve B and T lymphocytes lie mainly quiescent within the spleen and blood, as well as in other secondary lymphoid organs such as the liver and intestine (Figure 1A). In amphibians, the spleen functions as the primary and major secondary lymphoid organ. Naïve B and T lymphocytes each possess a unique and specific antigen receptor combination.

## Early naïve infection and constitutive defenses

The earliest stage of the infection process with Bd involves the likely chemotaxis of infectious zoospores toward the skin surface [Figure 1B, (11, 80, 81)], whereupon they encounter mucus and any associated constitutive defenses of the skin. We discuss these defenses in the context of chytridiomycosis below.

### Skin sloughing increases with chytridiomycosis and may physically reduce microbial burdens

Ecdysis or skin sloughing in ectotherms can function as a constitutive innate immune defense mechanism by physically removing skin microbiota (73, 82). Sloughing usually occurs soon after dark, approximately every 3 or 4 days depending on species, and the frequency of sloughing increases with ambient temperature (73). Abnormal sloughing is a clinical sign of chytridiomycosis and corresponds with an increased frequency of sloughing and the production of smaller shed pieces (3, 83, 84). This may be the result of sporangia initiating premature keratinization and cell death in infected epidermal cells, in concert with hyperplasia and stimulated epidermal turnover as observed by electron microscopy (77). Using infra-red video recordings, Ohmer et al. (84) found that while sloughing rate increased with infection, chytridiomycosis did not affect sloughing behavior, duration or rhythmicity, although diseased frogs typically did not eat the sloughed skin as they normally would. Independent of temperature, the extent to which increased sloughing reduces Bd infection loads varies depending on species' intrinsic susceptibility, and sometimes results in clearance of infection (85). As such, Ohmer et al. (84) hypothesized that skin sloughing may be both beneficial and detrimental in the face of chytridiomycosis (for example, by removing pathogens or symbiotic bacteria, and disrupting skin homeostasis). Of interest, the most resistant species they studied, *Limnodynastes tasmaniensis*, demonstrated increased sloughing rates at lower infectious burdens, which Ohmer et al. (85) suggested may indicate an effective induced defense response. Increased rates of sloughing in warmer environments, induced as an immune defense, or in association with behavioral fever (86, 87) may partly explain the improved clearance of Bd infection at higher temperatures (88).

### Natural mucosal antibodies (generated by innate-like B cells) may inhibit zoospores

Naïve frogs (not previously exposed to Bd) are unlikely to express specific mucosal antibodies that bind to and inhibit zoospores (89). However, as occurs in other vertebrates, natural antibodies may be present and may limit initial pathogen burdens. Natural antibodies are polyreactive against highly conserved pathogen epitopes. They are typically encoded by germline genes, and in mammals, they are produced by innate-like B cells (90–92). Little is currently known about their efficacy against Bd in amphibians.

### Lysozyme and other defensive enzymes may limit zoospore invasion

Lysozyme is a constitutively expressed antimicrobial enzyme found in body fluids and mucosal linings. Lysozyme from amphibian skin secretions has potent bactericidal activity (93, 94). Although typically considered an antibacterial enzyme, lysozyme has also been reported to possess antifungal properties (95, 96). Thus, amphibian lysozymes may have similar activity against pathogens such as Bd (11, 31, 97). To date there is little evidence for the role of lysozyme and other phagocyte- or keratinocyte-derived constitutive enzymes (e.g., antimicrobial lectins, secretory phospholipase A_2_) in defense against Bd. However, Rosenblum et al. (98) reported an increase in expression of lysozyme genes in the skin of infected *Xenopus tropicalis* at 21 days post exposure in a transcriptomic study. Grogan et al. (18) similarly identified upregulation of lysozyme C genes in the skin of *Litoria verreauxii alpina* throughout infection, although the efficacy of this response for limiting Bd infection is currently unknown.

### Amphibians produce a range of antimicrobial peptides with activity against Bd, and these likely play a role in defense

The antimicrobial peptides (AMPs) of vertebrate skin are typically small hydrophobic cationic peptides produced by serous glands that provide non-specific defense against pathogenic organisms (99, 100). Clarke (99) describes four main categories of defensive molecules including alkaloids, steroids, biogenic amines, and other peptides and proteins. The range and quantity of AMPs produced by amphibians is remarkable among vertebrates and has been the target of medical studies for decades, particularly for use in pharmaceutical applications [e.g., (101–103)]. The production of antimicrobial peptides can be induced and modulated by the presence of microbial flora (104) and chronic corticosteroid administration (105). Serous glands release antimicrobial peptides to the skin surface at a low continuous rate, however, mild activation of the sympathetic nervous system (such as alarm caused by a predator cue) is sufficient to stimulate the contraction of gland-associated muscle fibers and the release of larger quantities of AMPs to the skin surface (89, 106). It is unlikely that zoospore invasion alone would be sufficient stimulus to produce this response.

Through *in vitro* growth inhibition assays, many amphibian peptides and peptide mixtures (at concentrations likely to occur *in vivo*) have been found to inhibit the growth of various pathogens including Bd as well as other fungi [reviewed in (31, 107)]. Antimicrobial peptide defenses are considered reliable predictors of natural resistance of amphibians to chytridiomycosis. However, the efficacy of AMPs in defense against Bd appears to vary substantially by species and other factors, which may limit the value of AMP data for predicting and mitigating amphibian declines (108–113). These factors may include: (1) intrinsic peptide efficacy against Bd as demonstrated *in vitro*, (2) concentration, number and type of peptides produced, (3) rate and location of release to the skin surface in response to microbial pathogens, and (4) presence of host- and Bd-secreted proteases that may degrade AMPs (106, 114, 115). For example, depletion of AMPs led to increased infection probability in resistant amphibian species *Xenopus laevis* (89) and *Rana pipiens* (116), but not in *Pelophylax esculentus* and *P. lessonae* (117). Validation studies involving the functional modification of key AMP molecules are still needed to confirm these associations. Woodhams et al. (118) found that AMP expression differed between infected and uninfected wild-caught *Litoria serrata*, with infected frogs demonstrating reduced expression. However, they did not identify whether this was a cause or consequence of infection. Ribas et al. (119), Rosenblum et al. (120) identified AMP genes or precursors (including preprocareulein and cathelicidin) via microarray studies of the spleen and skin of frogs. More recently, comparing the anti-Bd activity of skin secretions collected from frogs before and after Bd emergence in Panama, Voyles et al. (15) found a significant increase in inhibitory efficacy post-emergence, consistent with an evolutionary shift in the host immune response. The mechanisms underlying this change in efficacy are unknown but may involve altered concentration and diversity of peptides or improved inhibitory function.

### The skin microbiome may inhibit zoospore colonization, and bioaugmentation may be an effective management strategy

Commensal microbial communities are present in the mucus layer on amphibian skin and may provide another mechanism of constitutive innate immunity against Bd via several mechanisms (32, 61, 121). For example, Meyer et al. (82) reported the cultivation of approximately 0.5–1.7 × 10^6^ bacterial colonies and 1.6–2.6 × 10^4^ fungal colonies per square cm of dorsal skin of *Rhinella marina*. Interestingly, detected microbial loads were much lower on ventral skin, despite being in more frequent contact with moist substrates than the dorsum. Many bacteria and fungi secrete antimicrobial compounds with repellant and growth-inhibitive properties against pathogenic microbes (122–124). Furthermore, microbiota may also compete directly with Bd, and may functionally change host immune responses (121).

Numerous epibiotic bacterial species isolated from amphibian skin are growth inhibitive for Bd *in vitro* (125, 126). For example, the bacterial species *Janthinobacterium lividum* has shown particular promise for the anti-Bd properties of its secreted metabolite violacein, at concentrations greater than 15 μM (127). In clinical Bd exposure experiments, both frogs (*Rana muscosa*) and salamanders (*Plethodon cinereus*) inoculated with *J. lividum* did not become infected (128), whereas depletion of bacteria resulted in high Bd infection intensities (129). This effect also extended to soil augmentation and environmental transfer of bacterial species to some amphibian hosts, thereby inhibiting Bd infection, at least temporarily (130, 131). Despite these promising results, outcomes in other species are mixed. For example, inoculation of Panamanian Golden frogs (*Atelopus zeteki*; extinct in the wild) with probiotic bacteria was not associated with improved survival (132, 133).

There is some evidence for population-level correlation between the presence of (and proportion of individuals harboring) anti-Bd bacterial species and population declines (134, 135), although not all studies corroborate these findings (136). The presence of diverse bacterial communities in addition to anti-Bd bacterial species on amphibians, and synergy (or antagonism) between bacteria and AMPs, will affect the degree of Bd growth inhibition as demonstrated *in vitro* (137–139). Other field studies have shown that skin microbial communities differ consistently between Bd-susceptible and resistant/tolerant species, as well as between sites with differing infection histories (121, 140). It is unclear from these studies whether identified bacterial communities promote Bd tolerance or resistance, or are the result of it (121). Further applied work should target probiotic candidate selection and trial bioaugmentation approaches (32, 141, 142).

## Innate immune defenses

If the invading Bd zoospores are not contained with constitutive host defenses, then they encyst upon the keratinized skin surface and attach via adhesive molecules (11, 77). Germination tubes are then sent through one or more cell layers (143, 144), injecting the contents of the zoospore cyst into the cytoplasm of deeper cells of the host epidermis, including the *stratum spinosum* and *stratum granulosum* [Figure 1B, (3)]. The intracellular location and process of injecting zoospore contents into deeper epidermal cells may permit Bd evasion of host immune surveillance, as has been described with other fungal pathogens (98, 145–147).

### Pattern recognition receptors detect common fungal structures and initiate inflammation, although there is currently limited data to assess their role in chytridiomycosis

In the absence of targeted immune evasion, an invading microbe should prompt host recognition. Mechanisms responsible for pathogen recognition induce innate then adaptive immune responses in the host via antigens either secreted, expressed on the pathogen cell surface, or processed after phagocytosis. These antigens often contain widely recognized structural moieties known as pathogen-associated molecular patterns (PAMPs) that are common among different groups of microorganisms. These PAMPs bind to host germline-encoded pattern recognition receptors (PRRs) expressed within or on cells of the innate immune system (macrophages, dendritic Langerhans cells) and nonprofessional immune cells (keratinocytes, fibroblasts) [reviewed in (35)]. In mammals, four classes of PRRs include: (1) Toll-like receptors (TLRs) and (2) C-type lectin receptors (CLRs) within cell membranes, (3) NOD-like receptors (NLRs), and (4) Retinoic acid-inducible gene (RIG)-I-like receptors (RLRs) within the cytoplasm of host cells (148). Binding of PAMPs by PRRs leads to an innate amplifying inflammatory cascade that varies depending on the initial signaling pathways involved. Binding of PRRs may induce signaling pathways and the release of cytokines [pathways such as nuclear factor kappa-light-chain-enhancer of activated B cells [NFκB], and mitogen-activated protein [MAP] kinase]. Binding may also stimulate phagocytosis and destruction of extracellular microorganisms, or cell-mediated cytotoxicity and apoptosis of infected host cells [reviewed in (35)]. Patin et al. (149) presents an extensive review of existing knowledge about fungal recognition in mammals via PRRs, and lists common fungal ligands including β-glucans, zymosan, mannose, phospholipomannan, unmethylated DNA, and chitin.

Little is known about PRRs and their interactions with fungal pathogens in amphibians, although genes homologous to mammalian PRRs are present in model amphibian genomes (e.g., *Xenopus* spp.). The expression pattern and inducibility of TLRs has been studied to some extent in *Xenopus* spp. and *Rana catesbeiana* (51, 150). To date there is little evidence for *early* upregulation of PRR-coding genes in frogs infected with any pathogens (including Bd), although this may be the result of a lack of early immune cell infiltration rather than a lack of constitutive recognition by nonprofessional immune cells *per se*. However, studies with detailed kinetic analysis appropriate for examining early innate immune responses in amphibians are currently lacking. For example, Grogan et al. (18) did not find any evidence of differentially expressed Dectin genes (key members of the CLR family for detecting common fungal pathogens) at any point during infection, compared with uninfected control frogs. However, several other putative PRRs (TLRs and NLRs) and their downstream signaling pathways were found upregulated in the skin of frogs during *late stage* infections (18, 98, 151–153). Representatives of these pathways included mannose-associated genes, fc receptor 5 genes, NFκB subunit genes, caspase 6, 7, and 10 analog genes, and MyD88 pathway genes (18). These results from across the several species studied suggest that putative PRR-encoding genes are present and are likely activated. Although, it is possible that the pathogen actively interferes with these pathways, such as the inhibition of NFκB detected by Rosenblum et al. (120). We currently lack sufficient evidence to determine the efficacy of the early innate immune response to Bd. The detected late gene activation of these downstream signaling pathways may alternatively be associated with cellular stress and trauma or secondary bacterial infections (151), producing damage associated molecular patterns (DAMPs) that are similarly recognized by PRRs and protease-activated receptors (PARs) (154). These findings are consistent with the results of Brem and Parris (155) who showed that toads were less likely to be become infected if the epidermis was scraped (causing erosions) prior to Bd exposure. Priming of the innate immune system in response to trauma (Bd-associated epidermal erosions) may contribute to amplification of the innate response, leading to an exacerbated late-stage response. Similar immune priming may have applications for infection mitigation strategies. The precise characteristics of pathogen recognition and signaling may be further elucidated by detailed kinetic studies.

### The alternative complement cascade may be important for early defense in resistant individuals

The complement system constitutes a set of receptors and soluble plasma proteins and enzymes with an important role in defense against pathogens. Complement activation stimulates a rapid cascade of molecular interactions triggered by bound antibodies and PAMPs that results in formation of the membrane attack complex (MAC) and plays a central role in innate defense against fungal infections [reviewed in (156)]. Bound complement components function to agglutinate extracellular pathogens and lyse their cell membranes, as well as attract phagocytes to the locality and enhance their phagocytosis of pathogens via opsonization. Although considered most capable at neutralizing extracellular pathogens, complement C3 binding prior to pathogen intracellularization can activate autonomous immunity within infected cells (via NFκB signaling), ultimately leading to cell destruction via apoptosis (157). The complement cascade may be activated via three mechanisms. The first mechanism is the classical pathway (triggered by antigen-antibody complexes, bacterial lipopolysaccharide, pentraxins such as C-reactive protein [CRP] and serum amyloid, etc.). A second mechanism is the alternative pathway (recognizes pathogen associated patterns or PAMPs). The third mechanism of activation is via the lectin pathway (recognizes carbohydrate structures via mannan-binding lectin [MBL], MBL-associated serine proteases [MASPs], and ficolins) [reviewed in (156)]. It is noteworthy that all elements of the complement system are well conserved across jawed vertebrates (158), such that with *Xenopus* spp. antiserum, it is possible to use purified mammalian complement to lyse amphibian red blood cells (159).

As an early and rapid defense response, examining the extent of complement activation may be critical for assessing the efficacy of the innate immune response. Several studies thus far have indicated early *downregulation* of complement pathway genes (98, 119, 120) in the infected skin of several susceptible species. In contrast, Grogan et al. (18) found that gene analogs associated with the alternative complement pathway (venom factor 1 and complement factor B) were upregulated from the early infection stage in a phenotypically more resistant population of *Litoria verreauxii alpina* frogs, but only upregulated at later infection stages in more susceptible populations (18). This is consistent with studies in a variety of species where complement pathway genes were predominantly upregulated in late-stage infection (151–153). These findings may indicate that the alternative complement pathway plays an important role in defense against Bd in more resistant individuals. However, Bd may downregulate or fail to activate the complement cascade in susceptible individuals, at least until the late infection stage (98). Further research on activation of the alternative complement pathway may provide genetic markers for resistance and opportunities for selective breeding or genetic engineering.

### There is limited data to assess cytokine upregulation in the crucial early-stage infection period

Cytokines are endogenous inflammatory mediators and include lymphokines (such as macrophage activating factor [MAF]), interleukins (ILs), tumor necrosis factors (TNFs), interferons (IFNs), transforming growth factors (TGFs), chemokines, colony stimulating factors (CSFs), polypeptide growth factors (GFs), and stress proteins [including heat shock proteins (HSPs)]. Pro-inflammatory cytokines can act on adjacent cells or distant cells via the systemic circulation to amplify the inflammatory cascade, attract leukocytes to the site of infection, activate pathways involved in blood coagulation, and promote tissue repair [reviewed in (35)].

Experimental studies performed on chytridiomycosis thus far have lacked sufficiently early time-points (i.e., 6–24 h post-exposure) and infection-targeted tissue sampling strategies to evaluate expression of putative cytokines or their encoding genes. However, gene expression studies sampling tissues between 3 and 8 days post exposure have reported limited evidence for upregulation of putative cytokine-encoding genes. These included IL-17A/F-like gene, calcineurin IL-2 inducible gene, HSPs, TNF associated factor (TRAF) and guanylate binding protein IFN inducible gene in spleens of *X. tropicalis* (98, 119), IFN and IL-associated genes in *Rana* spp. (120), and IL-1B, IL-17C, and IL-17E, TNFα, IFN and IFN-induced genes, granulocyte colony-stimulating factor, and several chemokine-associated genes in *Litoria verreauxii alpina* (18). In contrast, studies of *late*-stage infections demonstrated changes across the spectrum of cytokines (numerous IFNs, ILs, TNFs, and chemokines), with the most dramatic responses observed in skin tissues from the most susceptible individuals (18, 151, 152). Of particular interest, several gene expression studies on multiple species identified significant upregulation of numerous IFN-induced very large GTPase gene analogs throughout infection (18, 151, 160). Interferon-induced GTPase signaling is important for eliminating intracellular pathogens in epithelial cells via their sequestration and destruction within inflammasomes [thought to be especially important for defense against skin fungal pathogens (161)], and thus could be a key mechanism of cell-autonomous immunity in chytridiomycosis (162). From these results, it appears that putative cytokine pathways are active in host response to Bd, although we currently have insufficient data to evaluate their relevance in the immediate post-exposure period. The reported upregulated late-stage cytokine responses may instead represent immunopathology from a dysregulated and non-protective immune response (18, 151).

### There is limited evidence for innate leukocyte recruitment and infiltration throughout infection

Recruitment of leukocytes (immune effector cells) to the site of infection is a central component of the host immune response. Leukocytes of the innate immune system include circulating monocytes that differentiate into macrophages at the site of infection, polymorphonuclear phagocytes including neutrophils, eosinophils, and basophils, as well as natural killer cells and mast cells. These leukocytes contribute to recruit lymphocytes at the site of infection, amplify the inflammatory cascade, destroy extracellular pathogens via phagocytosis, and trigger apoptosis of damaged or infected host cells [reviewed in (35)].

The cellular immune response in chytridiomycosis appears inconsistent and is generally mild or *decreased* across species (48, 163). These studies largely compared skin and blood of Bd-infected and uninfected control frogs. For example, Woodhams et al. (111) found decreased circulating neutrophils and eosinophils, and increased numbers of basophils in infected adult *Litoria chloris* frogs. Davis et al. (164) and Peterson et al. (165) found increased circulating neutrophils and fewer eosinophils in infected *Rana catesbeiana* tadpoles and *Litoria caerulea* adults respectively [Peterson et al. (165) also found low circulating lymphocyte numbers [lymphopaenia]]. These results are consistent with a classic mammalian acute stress response [with the exception of the absence of lymphopaenia in the former study, (166)]. Young et al. (48) found lower circulating total white blood cell numbers in chronically infected adult *L. caerulea*, with overall impairment of responses on immune stimulation, and relatively higher neutrophil to lymphocyte ratios in infected frogs. These variable results may indicate that other unaccounted factors are playing a role (such as corticosteroid levels), or that species' responses differ.

The cellular immune response within skin tissue appears inconsistent from studies thus far performed. For example, histopathology has revealed a variable mild inflammatory response in 10–40% of skin sites, involving foci with macrophages and few neutrophils. This mild response is also often present near areas of ulceration suggesting a possible association with secondary bacterial infections (77, 151, 167, 168). No evidence of specific leukocyte-associated gene upregulation has been reported during early infections in gene expression studies performed thus far. However, during late-stage infection, Rosenblum et al. (98) found a mild increase in neutrophil-associated genes in the skin and liver of infected *Xenopus tropicalis* frogs, while Ellison et al. (151) and Grogan et al. (18) found predominantly increased expression of several macrophage and neutrophil associated genes. Taken together, these results indicate that an early leukocyte response is weak or lacking with Bd infection, and furthermore, that the late-stage response is inconsistent and likely insufficient to limit Bd infection (and may alternatively be associated with epidermal damage or secondary bacterial infection). This overall observed poor inflammatory response with Bd infection could be the result of inadequacy of innate immune activation with minimal cytokine-mediated leukocyte recruitment toward Bd. This could possibly be associated with immunoevasion and/or active suppression of immune responses by Bd. However, a limited innate immune response, particularly in late stage infections, may also be symptomatic of an inadequate or impaired *adaptive* immune response to Bd (discussed below).

## Adaptive immune response

The adaptive immune system provides a more specific defense against invading pathogens compared with the innate immune system, although it is slower to manifest initially. Amphibian pathogen-specific antibodies (IgY) are undetectable after initial ranaviral infection as is the case in mammalian response to primary infection with large DNA viruses (169). However, in mammals, antibody responses typically improve in efficacy upon subsequent exposures to the same pathogen [Figures 1C–F; reviewed in (35)], and studies performed in amphibians with Frog Virus 3 and Bd support this finding. Between two and three exposures to a pathogen over 4–6 weeks resulted in a detectable pathogen-specific IgY antibody response (89, 169, 170). These antibodies were detectable at 1 week after the last exposure, and peaked between 2 and 3 weeks. This means that the adaptive immune response may be most critical for infections that fail elimination by non-specific mechanisms of the innate immune response. In comparison with mammalian immune responses, the amphibian adaptive immune response is typically slower to manifest, and of lesser magnitude and efficacy. The adaptive immune system is also dependent on initial activation and co-stimulation by receptors and mediators of the innate immune system [reviewed in (35)]. However, as we have detailed in the previous sections, the innate immune response to Bd-infection appears somewhat inadequate, at least in the critical early stages of infection (first few days post exposure), and this may reduce the efficacy of the adaptive immune response.

### Key components of the adaptive immune response, pathogen specificity, and immunological memory

The primary components of the adaptive immune system include lymphocytes (T and B cells) and their respective mature effector forms responsible for enacting pathogen-specific cell-mediated immunity (cytotoxic or helper T cells) and humoral immunity (antibody-secreting plasma cells) together with secreted or membrane-bound antibodies (immunoglobulin). Unlike the germline-encoded components of the innate immune system, the adaptive immune system is characterized by unique antigen receptors. T and B cell receptors are generated when segments of immunoglobulin genes are rearranged by a unique process called somatic recombination. This can occur via V, D, and J (variable, diversity, joining) mechanisms, which require products from recombination-activating genes (RAGs). This gives rise to millions of naïve T and B cells during the development of an individual, with numerous distinct cell clones bearing unique surface receptors that together constitute the unique lymphocyte receptor repertoire of the host. Lymphocytes activated by antigen binding in combination with co-stimulatory molecules (Figure 1C) undergo clonal expansion and differentiation into their effector forms (Figure 1D). B cell receptors additionally undergo further changes after activation. This involves somatic hypermutation mediated by activation-induced cytidine deaminase (AICDA), which is accompanied by class switching during affinity maturation. During this clonal expansion process, large numbers of antigen-specific long-lived memory lymphocytes are produced (Figure 1E), and upon re-exposure to the pathogen or antigen, these memory lymphocytes respond more rapidly and effectively than the initial response (Figure 1F). Thus, not only is the adaptive immune system able to respond in a specific way to novel pathogens, but it *adapts* to those pathogens during the course of an infection. Assuming that the individual survives initial infection, a functioning adaptive immune response should increase in efficacy with subsequent exposures to a pathogen (or antigen) during an individual's lifetime (unlike the innate immune response), leading to the concept of immunization [or vaccination; reviewed in (35)].

### The role of PRRs, MHC and dendritic cells for activation of the adaptive immune response

Initial activation of the adaptive immune system involves the detection of pathogen-derived antigens by binding to PRRs expressed by host cells. Antigen binding stimulates endocytosis, degradation of the pathogen, and subsequent presentation of the antigen peptide on the cell surface via major histocompatibility complex (MHC) proteins. The two classes of MHC molecules, classes I and II, are expressed differently by cells of the body, and interact with different subsets of lymphocytes. MHC class I molecules are expressed by most nucleated somatic cells (for example, non-immune epithelial cells) and interact with CD8 cytotoxic T cells, leading to cytotoxicity and death of the host cell expressing antigen with the MHC class I molecule. MHC class I molecules are particularly important for recognition and elimination of intracellular pathogens via cell killing. MHC class II molecules are mainly expressed by professional immune cells such as dendritic cells and macrophages. These cells also recognize pathogens, but can present the antigen via the MHC class II molecules at the cell surface [reviewed in (35)]. Antigen binding then promotes differentiation of dendritic cells into antigen presenting cells (APC), and TNF-α stimulates APC migration to the spleen via the lymphatic or circulatory system where they contact lymphocytes with a variety of antigen-specific receptors (Figure 1C). MHC class II molecules bound to antigen on the surface of APCs interact with CD4 T helper cells in the presence of other co-stimulatory molecules, and their main function is to activate other immune effector cells (such as B cells; Figure 1C). Co-stimulatory molecules are expressed on APCs in response to mediators of the innate immune system (such as TLRs and NFκB), and they are essential for the activation of the naïve CD4 T helper cells.

### MHC genes associate with survival and are under positive selection, supporting the rapid evolution of resistance or tolerance to Bd

The diversity of MHC proteins expressed by cells is generated by polygeny (the presence of multiple interacting genes), allele codominance, and gene polymorphism [reviewed in (35)]. The evolution of MHC genes has been widely demonstrated to occur under selection by infectious diseases (171). Their inter-generational heritability (unlike T and B cell receptors) makes them important bridging elements between the innate and adaptive immune systems, and potential markers for selection of either resistance or tolerance to infection (14, 34, 172). Genes encoding MHC classes I and II molecules have been found to be upregulated throughout Bd infection, particularly within skin tissues (18, 34, 120, 151, 153). A variety of studies have demonstrated associations between characteristics of MHC alleles (allelic diversity, degree of heterozygosity, presence of certain alleles, presence of certain protein conformational elements) and degree of Bd susceptibility as it differs between species, populations and individuals (17, 20, 21, 173). Furthermore, several studies have demonstrated signals of positive selection at certain MHC loci in populations persisting with enzootic Bd, when compared with background levels of neutral genetic change (17, 20, 21). These findings suggest that certain MHC genes and alleles may play an important role in determining degree of Bd susceptibility, and that these are under directional selection for resistance or tolerance to Bd. However, recent recognition of the expansion of MHC class I-like genes in *Xenopus* spp. and presumably other ectothermic vertebrates [see Box 3; 64, 65] may require a revisit of some of the reported studies, especially transcriptomics. Indeed, MHC class I-like genes encode molecules with typical MHC primary structures but are polygenic and not or minimally polymorphic (66).

### Both cell-mediated and humoral immunity are likely important for defense against Bd

When lymphocytes are activated in the presence of peptidic antigen bound to MHC with appropriate co-stimulation, they proliferate by clonal expansion, differentiate into their effector type and migrate to the site of infection (Figure 1D). CD8 T lymphocytes stimulated by MHC class I-bound antigens differentiate into cytotoxic T cells that recognize and kill infected host cells. This form of cell-mediated immunity is likely to be especially important for intracellular pathogens such as Bd, as the most efficient means to eliminate the reproductive stage of the pathogen (zoosporangium) is to destroy infected host cells (31). However, B cells and their differentiated effector type (plasma cells) are likely to be similarly important for eliminating Bd. Unlike in mammals, amphibian B cells demonstrate phagocytic capabilities (58). B cells may also act as antigen-presenting cells for T helper cells, and their effector plasma cells produce antibodies (immunoglobulin, either membrane-bound or secreted) that may be capable of targeting and destroying extracellular pathogen stages such as zoospores and secreted toxins, as well as killing infected host cells. Antibodies target pathogens and kill infected host cells via a suite of mechanisms including (1) binding specifically with the epitope of the antigen and causing the antigens to agglutinate, inactivating them, (2) activating the classical complement cascade, leading to the membrane attack complex to lyse pathogens directly, and (3) antibody-dependent cell-mediated cytotoxicity (ADCC) involving tagging antigens for destruction by natural killer cells or phagocytes [reviewed in (35)]. Thus, both cell-mediated adaptive immunity (T cell elimination of the intracellular reproductive stage of Bd and B cell phagocytosis) and antibody-dependent humoral immunity [destroying zoospores and secreted products) are likely to be important for controlling Bd burdens (31)].

### There is limited evidence for an effective adaptive immune response to Bd infection

Although it has been suggested that herd immunity may protect populations if > 80% of frogs are immune (or resistant, through the effects of symbiotic bacteria) to Bd (134, 135, 174), there is currently little evidence to suggest that herd immunity operates in wild amphibian populations. Indeed, the existence of a herd immunity threshold relies on infection transmission being density-dependent, rather than frequency-dependent, as is expected for amphibian breeding aggregations (175). Similarly, herd immunity thresholds are unlikely to occur where the force of infection is unaffected by the presence of resistant individuals, as is the case for indirectly transmitted pathogens and those with multiple host species (or life-stages) with differing tolerance and susceptibility to Bd infection. Instead, the temporal patterns of enzootic Bd infection appear regulated by season and temperature rather than adaptive immunity in field populations (22). The few laboratory studies performed to date support these findings, suggesting limited activation of a protective adaptive immune response to chytridiomycosis. Young et al. (48) reported a decrease in total and IgY serum antibody responses [via anti-sheep red blood cells [SRBC] haemagglutination assay] in Bd-infected *L. caerulea* compared with uninfected frogs, while circulating numbers of lymphocytes were greatly reduced in infected frogs (48, 165). Results from histopathology of the skin showed only a mild response with foci of lymphocytes associated with regions of ulceration, or no evidence of lymphocytes (77, 163, 167, 168). In terms of gene expression results, Rosenblum et al. (98) found no change in lymphocyte markers or MHC genes in *X. tropicalis*. Results were similar in their second study on *Rana* spp. (120) despite mild upregulation of MHC class II genes in the skin during late-stage infection. Ribas et al. (119) found that adaptive immune genes were generally down-regulated in the spleen of *X. tropicalis* throughout infection. In contrast, other studies demonstrated upregulation of numerous adaptive immune genes associated with B and T lymphocytes, immunoglobulins and MHC genes, particularly in skin tissues at late stages of infection (18, 151, 160, 176). However, a countering signal of downregulated T cell associated genes was also detected in several studies (18, 151, 152). These conflicting results indicate a more complex set of interactions operating within the adaptive immune system, which may be associated with the different temperatures at which animals were exposed or housed as well as the timing of collection of samples. The latter finding of downregulated T cell responses is particularly important and will be discussed in detail in its own section below.

Immunization against Bd was suggested early on as a management strategy for chytridiomycosis (177) given the highly successful examples from humans and domestic animals (178). Studies reported by Rollins-Smith et al. (31) and Ramsey et al. (89) attempted to immunize *Xenopus laevis* frogs against chytridiomycosis via an intraperitoneal injection with heat-killed Bd. They found promising results with a high-titer pathogen-specific IgM and IgY serum antibody response in the immunized frogs at 14 days post final immunization. In another experiment, Bd-binding mucosal antibodies (IgM, IgY, and IgX) were demonstrated after repeated exposure to Bd (89). These results are supported by the finding of upregulated immunoglobulin genes in re-exposed *Atelopus zeteki* frogs, suggesting production of memory lymphocytes (151). Furthermore, X-irradiation of frogs to impair T cells increased Bd infection loads in *X. laevis* (89). In contrast, a repeat experiment with killed-Bd injections into the dorsal lymph sac (days 0 and 14) and peritoneum (day 28) of *X. laevis* followed by splenocyte culture with Bd showed generally weak lymphocyte proliferation in comparison with samples cultured with phytohaemagglutinin (PHA) alone (31). In another experiment, young boreal toads (*Bufo boreas*) were immunized following a similar protocol and then exposed to Bd, however there was no evidence for a difference in survival between the immunized and sham-injected exposed frogs, suggesting that the immunization had not been successful in stimulating protective adaptive immunity in the young toads (31). Stice and Briggs (179) immunized *Rana muscosa* with formalin-killed Bd in combination with adjuvants [saline, Freunds Complete [FCA], and Incomplete Adjuvant [FIA]] by injection into the dorsal lymph sac and found no differences in the proportion of frogs infected nor time to infection. A study by Cashins et al. (180) did not detect any evidence for a protective effect of prior infection on re-exposure in *Litoria booroolongensis*. However, a study in *B. boreas* by Murphy et al. (181) found that previously exposed frogs survived slightly longer if they had a dry habitat option upon re-exposure. A study by McMahon et al. (182) found that multiple prior exposures to Bd slowed the rate of progression of chytridiomycosis, although this finding may instead be associated with repeated innate immune priming through trauma (155). The variable results of these studies may be associated with differing routes of immunization or dose-rates of Bd exposure. Furthermore, these results suggest that although the adaptive immune system may be activated during Bd infections in some species, the capacity for a robust and protective adaptive response appears limited, which may be associated with Bd-induced suppression (discussed below).

## Hot topics: stress, immunosuppression and immunopathology in chytridiomycosis

### Limited evidence that stress predisposes hosts to chytridiomycosis via corticosterone responses

There is no evidence to suggest that immunosuppression is necessary to predispose amphibians to chytridiomycosis epizootics, particularly with numerous observations of disease emergence in abundant species in undisturbed naïve localities (3, 163, 183). Furthermore, signs indicative of generalized immunosuppression, such as secondary bacterial infections, appear to be largely lacking (8, 48, 88). However, stress-induced immunosuppression may play a role particularly in the infection of more resistant individuals and species [reviewed in (30)]. The extent to which environmental stressors and corticosterone mediate chytridiomycosis and its effects on amphibians is currently unclear. Environmental stressors (poor nutritional status, high densities and exposure to predator cues) have been putatively linked with elevated corticosterone and reduced immune capacity in some tadpole studies (184, 185), although it is unknown whether corticosterone is a direct mediator of these effects. Elevations in corticosteroids have been demonstrated to have a range of detrimental effects on the immune system of frogs, including inhibiting the humoral response, and reducing both numbers and viability of circulating lymphocytes [reviewed in (30)]. Indeed, exogenous application of corticosterone was found to increase Bd infection abundance in adult amphibians (105, 186), but only had sublethal effects on tadpoles (187). Gabor et al. (188) inhibited corticosterone synthesis (using metyrapone) and found that this did not prevent Bd-associated reductions in mass, although it did increase Bd loads. They concluded that the adverse effects of Bd on growth were not mediated by corticosterone.

In the field, non-invasive measures of corticosterone in free-living populations of tadpoles revealed that corticosterone levels correlated both with Bd infection and altitude, and that infections with a more virulent strain of Bd (BdGPL) led to higher corticosterone release (189, 190). Measuring urinary corticosterone, Graham et al. (191) and Kindermann et al. (192) similarly found higher levels in infected frogs as well as frog populations at higher altitudes. Furthermore, Peterson et al. (165) measured plasma corticosterone and found that diseased frogs (showing clinical signs of chytridiomycosis) demonstrated higher corticosterone levels than subclinically infected frogs. Thus, from current evidence it appears that elevated corticosterone correlates with infection *in situ*, and both predisposes to chytridiomycosis, and is a result of infection. However, the link between putative environmental stressors and elevated corticosterone is less robust, and elevated corticosterone does not appear to mediate the sublethal effects of chytridiomycosis (growth and mass).

### *batrachochytrium dendrobatidis* suppresses lymphocyte responses in susceptible individuals

Throughout this review, we have synthesized the results of numerous studies and highlighted the lack of a generally robust and protective immune response to Bd infection. We considered the epidemiology, general degree of inflammation, as well as markers of the innate immune response during early infection stages, and the adaptive immune response during late infection stages. This observed apparent lack of immune response may be the result of either (1) the failure of the host to recognize Bd as a pathogen, through low inherent antigenicity (possibly due to intracellular localization), immunoevasion, or masking antigens, or (2) Bd-induced immunosuppression or downregulation of key immune responses necessary for a protective immune response (145). Both of these mechanisms may occur in parallel in chytridiomycosis. For example, PRRs are generally not upregulated in early infection, suggesting a possible lack of pathogen recognition, whereas T cell responses appear actively suppressed or inhibited, as are complement-associated pathways.

Current evidence supports a specific role for Bd-induced immunosuppression, detected first via skin histopathology (77) and general immune function measures (31, 48), and corroborated via gene expression data (18, 98, 119, 120, 151, 153). Further experimental work has characterized at least one mechanism by which this might occur, via soluble Bd-secreted factors. Fites et al. (193) demonstrated that soluble factors released by Bd zoosporangia inhibited proliferation and/or caused apoptosis of T cells *in vitro*. For this work, they used *in vitro* immune experiments involving the proliferation of splenic lymphocytes (from *X. laevis* and *R. pipiens*) in culture. Interestingly, they found that macrophage phagocytosis was not similarly affected. Another study investigated apoptosis (via TUNEL and caspase assays) and found that programmed cell death was positively associated with infection load and morbidity (194). They speculated that apoptosis may thus be a pathogen virulence mechanism. *In vivo* studies also revealed immune inhibition activity associated with Bd supernatants by measuring delayed-type-hypersensitivity responses (33). Rollins-Smith et al. (195) then went on to characterize two metabolites (methylthioadenosine and kynurenine) produced by Bd that are capable of inhibiting lymphocyte proliferation and survival *in vitro*.

### Late stage immunopathology characterizes infections in susceptible individuals

Despite relative Bd-associated immunosuppression, several gene expression studies on a variety of amphibian species have demonstrated that susceptible individuals express both greater number and variety of dysregulated immune genes during late stage infections than more resistant individuals (18, 151, 152). This negative correlation between extent of immune response and degree of phenotypic susceptibility suggests that susceptible individuals may mount massively dysregulated and non-protective immune responses. This immunopathology is likely associated with DAMPs induced late in infection as the pathogen damages skin cells in order to release subsequent generations of zoospores. Such a dysregulated response may rapidly disrupt cellular homeostatic mechanisms. Indeed, recent metabolomics findings support this hypothesis by demonstrating the significant depletion of the “immune nutrient factor,” alpha-ketoglutarate and its associated metabolite glutamate in severely infected animals (196, 197). This metabolic dysregulation has carry-on effects on numerous other aspects of cell homeostasis, particularly cellular energy metabolism (alpha-ketoglutarate is a key intermediate of the Krebs cycle). Furthermore, *in vitro* and gene expression studies suggest massive disruption of homeostatic mechanisms involved in epithelial stability, water and ion transport and musculoskeletal functions in susceptible individuals (18, 41, 98, 120, 151–153, 176). Therefore, immunopathology within susceptible amphibian species may not only cause their immune responses to be ineffective at eliminating the pathogen, but it may contribute to host morbidity and mortality due to the extensive disruption of cellular homeostasis and consumption of energy resources.

## Recommendations for future work

Despite two decades of research on chytridiomycosis, we still have only a limited understanding of the amphibian immune response to chytridiomycosis, and there is much to be discovered that may assist with disease mitigation. While continued support for existing approaches is essential, improving our capacity for amphibian immunological research will require: (1) the selection of an appropriate Bd-susceptible model species that could be bred to a MHC defined inbred strain (traditional amphibian models, *Xenopus* spp., are not sufficiently susceptible), (2) the development of a suite of taxon-specific affinity reagents (such as antibodies) for detection and imaging of pathogen-associated or host immune molecules of interest, and (3) the isolation or transgenic development of cell lines (including immune cells and skin explants) for *in vitro* functional assays.

A suite of conventional and emerging immunological methods from the fields of human and comparative immunology may be adapted for further study of amphibian chytridiomycosis. These methods enable the detection, quantification, isolation, functional evaluation, examination of signaling pathways, and localization of specific molecules of interest from homogenates, subcellular compartments (via biochemical fractionation), cells (separated by fluorescent-activated cell sorting [FACS] and flow cytometry), blood or tissues. For example, bioassays such as enzyme-linked immunosorbent assay (ELISA), measure the presence of various molecules (such as antibodies or antigens) via enzyme or ligand binding. Other bioassays may detect the presence and quantity of specific DNA (southern blot or qPCR), RNA (northern blot or RT-PCR), proteins (western blot) or their post-translational modifications (eastern blot). Flow cytometry enables the analysis of immune cells and their products, and cell sorting for proliferation and viability studies. Modern high-resolution imaging technologies include light microscopy combined with flow cytometry or standard labeling techniques with antibodies (immunohistochemistry and immunocytochemistry) or other stains, as well as electron-microscopy.

Several preliminary exploratory systems biology studies have been reported in this review, for example, employing transcriptomics for gene expression and metabolomics for metabolite accumulation (152, 196). However, there are emerging approaches using high-throughput technologies such as next generation sequencing and mass spectrometry that still have unrealized potential for the study of amphibian chytridiomycosis (such as whole exome sequencing, proteomics, secretomics, and fluxomics). Importantly, further research using these emerging technologies would benefit from considering a broader temporal range in samples from experimental animals. In particular, experiments that compare the immune response in the very early infection period immediately post-exposure with the response later during infection would shed important light on initial susceptibility and within-host pathogen recognition and signaling dynamics. There is also potential for the use of microfluidics for single-cell-targeted approaches. Mass spectrometry and associated technologies [high performance liquid chromatography (HPLC) and nuclear magnetic resonance (NMR)] permit the high-throughput separation, identification and quantification of molecules of interest in a mixture. Combination techniques may permit high-dimensional data from these high-throughput technologies, such as the single-cell resolution of numerous cellular parameters over millions of cells via mass cytometry. Chromosome conformation capture may permit the identification of regulatory elements for immune genes of interest, and transgenic technologies may enable improved functional validation of the role of such genes and their translated protein products. These approaches include gene knock-in and knock-out on the pathogen and other organism cell lines (such as host immune cells), and include gene silencing (RNA interference), lentivectors, transposons and CRISPR/Cas9 genome editing. Indeed, some of these approaches may be used to advance therapeutic outcomes also. For example, recombinant Bd proteins or Bd genes introduced via vector may improve results in immunization trials compared with techniques already tried.

## Conclusions

In summary, we have provided an overview of the major aspects of the amphibian host immune response to chytridiomycosis, and how they differ from an expected efficacious immune response. Importantly, we highlighted an observed discord between the extent and efficacy of the response to chytridiomycosis comparing resistant and susceptible individuals. These findings suggest that resistant individuals likely possess more effective constitutive defenses (such as AMPs and symbiotic bacteria), and/or may mount a more effective innate immune response early in infection, combined with avoiding Bd-induced immunosuppression of their adaptive responses. Conversely, constitutive and innate defenses of individuals that succumb to chytridiomycosis are likely limited in their overall efficacy. Although their late-stage immune response may be characterized by exacerbated immune gene transcription, these responses likely constitute immunopathology, and may be ineffective due to pathogen-suppression of lymphocyte pathways. Indeed, severe immune dysregulation may contribute to a mortality outcome. Hence a combination of factors likely contributes to amphibian susceptibility to chytridiomycosis, rather than the presence or absence of any one immune mechanism or gene. This is particularly important when comparing potential factors conferring resistance or tolerance between distantly related amphibian taxa.

Our review has highlighted numerous gaps in current knowledge, particularly concerning: (1) mechanisms of initial pathogen detection and possible immunoevasion by Bd, (2) degree of activation and efficacy of the innate immune response, (3) the unexpected absence of innate leukocyte infiltration, (4) the relative importance of B and T cell responses for pathogen clearance, (5) the capacity and extent of immunological memory, (6) specific mechanisms of pathogen-induced immunosuppression, and (7) the role of immunopathology in pathogenesis. These aspects would benefit from further empirical study using the techniques we have discussed above. This also leaves us with an unanswered question for amphibian conservation management: can we manipulate the immune machinery of the host to improve resistance or tolerance both within individuals (immunization), and across populations through generations (evolution or assisted selection)? It is important to recognize that management approaches should be considered on two time-scales; (1) securing species in the short-term, and (2) developing long-term sustainable solutions (12). As we have reported earlier in this review, evidence is emerging that evolution of resistance and tolerance may be leading to recovery of some affected frog populations and communities (15). There is, as yet, limited proof of concept for strategies that might accelerate these evolutionary processes. However, immunological research remains a promising avenue for amphibian conservation management, in light of the dramatic advances achieved in the human medical field in recent years.

## Author contributions

LG reviewed the literature and wrote the first draft. All authors contributed to revisions of the manuscript drafts.

### Conflict of interest statement

The authors declare that the research was conducted in the absence of any commercial or financial relationships that could be construed as a potential conflict of interest.
